# A Comparative Analysis of Artificial Intelligence Platforms: ChatGPT-4o and Google Gemini in Answering Questions About Birth Control Methods

**DOI:** 10.7759/cureus.76745

**Published:** 2025-01-01

**Authors:** Erhan Muluk

**Affiliations:** 1 Obstetrics and Gynaecology, Anatolia Hospital, Antalya, TUR

**Keywords:** artificial intelligence, birth control methods, chatgpt-4o, contraception, google gemini, health information

## Abstract

Background

Birth control methods (BCMs) are often underutilized or misunderstood, especially among young individuals entering their reproductive years. With the growing reliance on artificial intelligence (AI) platforms for health-related information, this study evaluates the performance of ChatGPT-4o and Google Gemini in addressing commonly asked questions about BCMs.

Methods

Thirty questions, derived from the American College of Obstetrics and Gynecologists (ACOG) website, were posed to both AI platforms. Questions spanned four categories: general contraception, specific contraceptive types, emergency contraception, and other topics. Responses were evaluated using a five-point rubric assessing Relevance, Completeness, and Lack of False Information (RCL). Overall scores were calculated by averaging the rubric scores. Statistical analysis, including the Wilcoxon Signed-Rank test, Friedman test, and Kruskal-Wallis test, was performed to compare metrics.

Results

ChatGPT-4o and Google Gemini provided high-quality responses to birth control-related queries, with overall scores averaging 4.38 ± 0.58 and 4.37 ± 0.52, respectively, both categorized as "very good" to "excellent." ChatGPT-4o demonstrated higher scores in the lack of false information, based on descriptive statistics (4.70 ± 0.60 vs. 4.47 ± 0.73), while Google Gemini outperformed in relevance, with a statistically significant difference (4.53 ± 0.57 vs. 4.30 ± 0.70, p = 0.035, large effect size). Completeness scores were comparable (p = 0.655). Statistical analyses revealed no significant differences in overall performance (p = 0.548), though Google Gemini demonstrated a potential trend of stronger performance in the "Other Topics" category. Within-model variability showed ChatGPT-4o had more pronounced differences among metrics (moderate effect size, Kendall’s W = 0.357), while Google Gemini exhibited smaller variability (Kendall’s W = 0.165). These findings suggest that both platforms offer reliable and complementary tools for addressing knowledge gaps in contraception, with nuanced strengths that warrant further exploration.

Conclusions

ChatGPT-4o and Google Gemini provided reliable and accurate responses to BCM-related queries, with slight differences in strengths. These findings underscore the potential of AI tools, in addressing public health information needs, particularly for young individuals seeking guidance on contraception. Further studies with larger datasets may elucidate nuanced differences between AI platforms.

## Introduction

The global population is projected to increase from 7.7 billion in 2019 to 8.5 billion by 2030 (10%), 9.7 billion by 2050 (26%), and 10.9 billion by 2100 (42%) [1]. This growth highlights the pressing need to address the unmet demand for family planning, which affects 20%-58% of individuals, especially in low- and middle-income countries [2]. It has been revealed that younger women, in particular, face significant gaps in reproductive knowledge; 65.5% of high school students lacked adequate sexual and reproductive health literacy [2,3]. In 2019, adolescents aged 15-19 experienced 21 million pregnancies annually, with half unintended, resulting in approximately 12 million births [4].

When faced with an unintended pregnancy, a woman has two primary options. She may choose to continue the pregnancy; however, unintended pregnancies are significantly linked to higher risks of maternal depression during pregnancy and postpartum, interpersonal violence, preterm birth, and low infant birth weight [5].

Alternatively, she may seek to terminate the pregnancy, with abortion being the most commonly utilized procedure. However, this option carries significant risks, as global estimates from 2010 to 2014 indicate that 45% of all induced abortions are unsafe [6]. In regions where unsafe abortions are common, maternal death rates can exceed 200 per 100,000 abortions [7]. According to the World Health Organization (WHO), adolescent mothers, aged 10-19 years, face greater risks, including complications such as eclampsia, puerperal endometritis, and systemic infections. Their babies are also more likely to experience low birth weight, preterm birth, and severe neonatal conditions. Therefore, preventing adolescent pregnancies and reducing related mortality and morbidity are critical for achieving global goals related to maternal and newborn health [4].

Addressing these challenges requires improving access to birth control methods (BCMs). If all women in developing countries used contraceptives, it could prevent 22 million unplanned births, 25 million induced abortions, 15 million unsafe abortions, 90,000 maternal deaths, and 390,000 children from losing their mothers [8]. This demonstrates the transformative potential of family planning in improving health outcomes and reducing the global burden of unintended pregnancies and their associated risks.

Young individuals may encounter barriers to obtaining contraception information, such as limited access to healthcare services, the demanding schedules of providers, or reluctance to discuss sensitive topics.

This critical subject could benefit from alternative approaches, with artificial intelligence (AI) emerging as a promising tool to bridge these gaps. AI platforms, designed to source and synthesize information from extensive databases and authoritative resources, provide an advanced alternative to traditional search engines for accessing reliable health information.

AI platforms enhance patient care and support physicians by providing valuable data for decision-making [9]. In gynecology, they aid in diagnosis, optimize treatment plans, and improve surgical outcomes, contributing to superior patient care [10]. Additionally, they effectively address patient questions across various medical fields [11-16]. Tools like ChatGPT and Google Gemini utilize sophisticated language models to answer health-related questions using diverse sources, including medical databases, research studies, and health websites [17,18]. By providing accessible and reliable information, conversational AI addresses barriers individuals face when seeking contraception guidance.

This study aimed to evaluate the responses of two conversational AI systems, ChatGPT-4o and Google Gemini, to a series of questions derived from the most frequently asked questions for teens about birth control, derived from the American College of Obstetricians and Gynecologists (ACOG) webpage [19]. The goal was to assess the effectiveness of these systems in providing accurate and personalized contraception information to young individuals who may lack access to professional medical care. The study tested two primary hypotheses: first, that the responses provided by ChatGPT-4o and Google Gemini would demonstrate high performance, being more than satisfactory in addressing the questions, versus the null hypothesis that their responses would not meet this standard; and second, that there would be significant differences in the quality and relevance of the responses provided by ChatGPT-4o and Google Gemini due to differences in their underlying technologies, training data, and model architectures, versus the null hypothesis that no significant differences would exist between the two AI systems.

## Materials and methods

Study design

This study employed a mixed-methods approach, combining qualitative and quantitative elements to address the research objectives comprehensively. Guided by the METRICS checklist, designed for AI-based evaluations, the methodology ensured rigor and thoroughness [20]. As the study focused on evaluating conversational AI interactions without involving human participants, formal ethical approval was not required.

Models used

Two widely utilized conversational AI systems, ChatGPT-4o and Google Gemini, were selected as representative examples of advanced AI technology. While Google Gemini was publicly available, access to ChatGPT-4o required a subscription during the study period.

Evaluation of the generated content

An assistant presented the predefined questions to the AI models, recorded all interactions, and anonymized the data by assigning fictional names to the models. The responses were then evaluated collaboratively by the author, a specialist in obstetrics and gynecology, and a nurse with significant expertise in family planning. This collaboration ensured the robustness and reliability of the results, combining clinical and practical perspectives.

The assessment criteria of the AI-generated answers were adapted from a standardized tool specifically designed to evaluate the quality of health information generated by AI-based models [21]. The responses were evaluated using three metrics: Relevance, Completeness, and Lack of False Information (RCL). Each metric, outlined in Table 1, was scored on a scale from 1 to 5, where 1 represents the lowest performance and 5, the highest.

**Table 1:** Criteria used to evaluate AI responses AI: Artificial intelligence

Criteria	Score	Description
Relevance	1	The information is completely unrelated to the topic or purpose; entirely off-track.
	2	Some connection to the topic, but lacks key points; includes irrelevant or tangential information.
	3	Moderately relevant; includes both pertinent and extraneous information, affecting overall usefulness.
	4	Highly relevant to the topic, with only minor instances of less pertinent information.
	5	Fully relevant to the topic; all information directly supports the purpose and context.
Completeness	1	The information is missing essential details, providing an incomplete understanding of the topic.
	2	The information includes some important details but leaves out several significant aspects.
	3	The information is generally complete but may omit minor details that slightly limit comprehensiveness.
	4	The information is very complete, with negligible omissions.
	5	The information is exhaustively complete, leaving no significant gaps.
Lack of false information	1	Contains excessive false information that is highly misleading and undermines trust.
	2	Includes some false or misleading information; key aspects misquoted or misrepresented, reducing reliability.
	3	Generally accurate, but some minor inaccuracies may cause slight confusion or uncertainty.
	4	Largely accurate; only minor and insignificant inaccuracies present that do not detract from overall reliability.
	5	Completely accurate and trustworthy, contains no false or misleading statements; fully aligned with reliable sources.

Timing of testing, transparency of the data source

AI models were tested on November 9, 2024; conversations were recorded and archived in the public data repository Zenodo [22].

Range of tested topics, randomization of selecting the queries

The topics tested were sourced from the ACOG women's health webpage, ensuring comprehensive and systematic inclusion of all related questions frequently asked by teens [19]. Since all topics were included, randomization in query selection was not required.

Individual factors in selecting the queries, count of queries

No individual factors influenced the selection of queries, as they were directly taken from the source. All 30 questions were utilized in the analysis.

Specificity of prompts/language

Prompts were designed to reflect everyday language commonly used by young people seeking health information, starting with the phrase, "I am a young person who wants to ask a question about birth control. My question is this..." and concluding with a query about BCMs. Both systems were tested in their default settings to ensure consistent and replicable results. As ChatGPT-4o and Google Gemini do not retain information from previous interactions, each inquiry began as a new conversation, eliminating potential learning or feedback loops. All prompts and responses were conducted in Turkish. Table 2 presents the categorized list of questions posed to the AI platforms about BCMs.

**Table 2:** Categorized questions posed to AI platforms about birth control methods IUD: Intrauterine device; AI: Artificial intelligence

Category	Questions
Contraception in general	What should I pay attention to when choosing a birth control method?
	Do I need a pelvic exam to get a prescription for birth control pills?
	What are the best birth control methods to prevent pregnancy?
	Which birth control methods also protect against sexually transmitted diseases?
	How does hormonal birth control work?
	Where can I find free or low-cost birth control?
Specific contraceptive types	What is a birth control pill?
	What is a skin patch?
	What is a vaginal ring?
	What is a birth control injection?
	What is an implant?
	What is an IUD?
	How do IUDs work?
	What are barrier methods?
	What is spermicide?
	What is a condom?
	What is a diaphragm?
	What is a cervical cap?
	What is a contraceptive sponge?
Emergency contraception	What is emergency contraception?
	What are the types of emergency contraception?
	How is a copper IUD used for emergency contraception?
	What are the types of emergency birth control pills?
	Where can I get emergency birth control pills?
	Where can I find more information about emergency contraception?
Other topics	What should I consider when deciding to have sex?
	How does pregnancy occur?
	What is the best way to talk to my partner about condoms?
	Do I need my parents' permission for birth control?
	If I use their insurance for birth control, will my parents find out?

Statistics

Statistical analysis was performed using IBM SPSS Statistics for Windows, Version 29.0.2.0 (Released 2023; IBM Corp., Armonk, NY, USA), with a significance set at p < 0.050. Raw scores from the assessment criteria were used, and overall scores for each question were calculated by averaging the scores of the three criteria. The overall scores were later categorized as follows: 1-1.79 (poor), 1.80-2.59 (satisfactory), 2.60-3.39 (good), 3.40-4.19 (very good), and 4.20-5.00 (excellent).

Given the ordinal nature of the data, non-parametric tests were employed. The Wilcoxon Signed-Rank test compared paired metrics between ChatGPT-4o and Google Gemini, the Friedman test assessed within-model variability across metrics, and the Kruskal-Wallis test evaluated performance differences across question categories.

## Results

**Figure 1:**
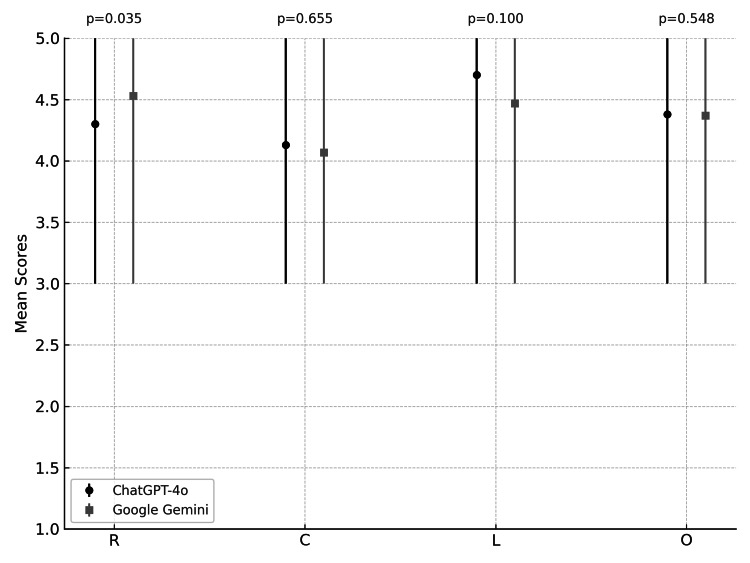
Score distribution of ChatGPT-4o and Google Gemini R: Relevance; C: Completeness; L: Lack of false information; O: Overall scores

The statistical evaluation of responses to 30 birth control-related questions provided by two AI platforms was summarized in Table 3. ChatGPT-4o exhibited slightly higher mean scores for "completeness" and "lack of false information," while Google Gemini excelled in "relevance." Overall, the performance of both platforms was comparable, with minor differences in standard deviations indicating consistency in response quality across the metrics. These findings suggest that both platforms provide reliable responses for health-related queries, with ChatGPT-4o marginally leading to "lack of false information." The results indicate that ChatGPT's "lack of false information" metric stands out positively compared to its "completeness" metric, emphasizing the model's focus on accuracy over completeness (Table 3; Figure 1).

**Table 3:** Descriptive statistics of ChatGPT-4o and Google Gemini performance on birth control related questions SD: Standard deviation; CI: Confidence interval

Metric	ChatGPT-4o mean ± SD	ChatGPT-4o CI (95%)	Performance (ChatGPT)	Google Gemini mean ± SD	Google Gemini CI (95%)	Performance (Google Gemini)
Relevance	4.30 ± 0.70	(4.05, 4.55)	Excellent	4.53 ± 0.57	(4.33, 4.74)	Excellent
Completeness	4.13 ± 0.73	(3.87, 4.39)	Very good	4.07 ± 0.74	(3.80, 4.33)	Very good
Lack of false information	4.70 ± 0.60	(4.49, 4.91)	Excellent	4.47 ± 0.73	(4.21, 4.73)	Excellent
Overall score	4.38 ± 0.58	(4.17, 4.59)	Excellent	4.37 ± 0.52	(4.18, 4.55)	Excellent

**Figure 2:**
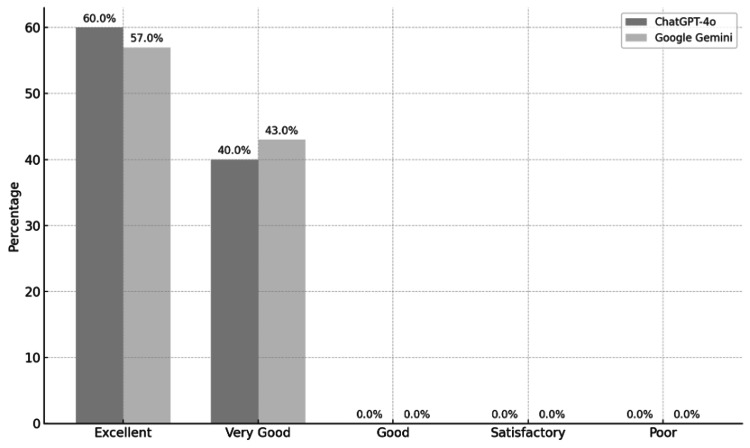
Category distribution of ChatGPT-4o and Google Gemini according to overall scores Poor: 1-1.79; Satisfactory: 1.80-2.59; Good: 2.60-3.39; Very good: 3.40-4.19; Excellent: 4.20-5.00

The majority of overall scores for both platforms fell into the "excellent" category (ChatGPT-4o: 60%, Google Gemini: 57%), followed by "very good" (ChatGPT-4o: 40%, Google Gemini: 43%). No responses for either platform were classified as poor, satisfactory, or good. These findings suggest that both ChatGPT-4o and Google Gemini consistently provide high-quality responses to birth control-related questions, with slight variability favoring ChatGPT in the highest classification (Figure 2).

Both the Kolmogorov-Smirnov test and the Shapiro-Wilk test yielded p-values < 0.050 for all metrics, indicating that none of the distributions meet the assumption of normality. So, the Wilcoxon-Signed-Rank test was utilized. The analysis revealed that while Google Gemini showed better performance in relevance over ChatGPT-4o, the AI platforms performed comparably on completeness, lack of false information, and overall scores. This suggests that although relevance is a differentiating factor, both platforms provide a similar experience (Table 4).

**Table 4:** Wilcoxon Signed-Rank test results for comparison between ChatGPT-4o and Google Gemini metrics *Statistically significant

Metric	Z-value	p-value (two-tailed)	Effect size (r)	Effect size interpretation
Relevance	-2.111	0.035*	-0.385	Large
Completeness	-0.447	0.655	-0.082	Small
Lack of false information	-1.645	0.100	-0.300	Large
Overall score	-0.601	0.548	-0.110	Medium

The Kruskal-Wallis test was conducted to determine whether the overall scores for ChatGPT-4o and Google Gemini differed significantly across the four question categories. While there were differences in mean ranks across categories for both models, they were not statistically significant. In the "Other Topics" category, both ChatGPT-4o (22.00) and Google Gemini (23.20) had higher mean ranks than in other categories. This implied stronger performance in this category relative to others. The p-values indicated no significant differences across categories, but, for Google Gemini, the trend (p = 0.067) suggests that, with more data (larger sample size), "Other Topics" might demonstrate a significant difference from some of the other categories (Table 5).

**Table 5:** Kruskal-Wallis test results for overall scores across question categories H: Kruskal-Wallis test statistic; df: degrees of freedom; p-value (asymptotic significance): probability value indicating statistical significance

Overall score	Categories	N	Mean rank	Kruskal-Wallis H	df	p-value (asymptotic significance)
ChatGPT-4o	General contraception	6	15.67	4.797	3	0.187
	Specific contraceptive types	13	12.35	-	-	-
	Emergency contraception	6	16.75	-	-	-
	Other topics	5	22	-	-	-
Google Gemini	General contraception	6	9.42	7.148	3	0.067
	Specific contraceptive types	13	15.58	-	-	-
	Emergency contraception	6	15	-	-	-
	Other topics	5	23.2	-	-	-

The Friedman test was used to examine how consistently ChatGPT-4o and Google Gemini performed across three key metrics: RCL. For ChatGPT-4o, the test revealed a statistically significant difference in performance across the metrics (Chi-square = 21.415, df = 2, p < 0.001). Pairwise comparisons using the Bonferroni correction showed that completeness differed significantly from lack of false information (p = 0.024), while no significant differences were observed between relevance and completeness (p = 1.000), or between relevance and lack of false information (p = 0.212). These results suggest that, while ChatGPT-4o performed consistently on most metrics, completeness was notably distinct from lack of false information.

Similarly, the Friedman test for Google Gemini also indicated significant differences among the metrics (Chi-square = 9.912, df = 2, p = 0.007). However, pairwise comparisons revealed no significant differences between completeness and lack of false information (p = 0.184), or between lack of false information and relevance (p = 1.000). A marginal difference was observed between completeness and relevance (p = 0.085), but it did not reach statistical significance after adjustment. This indicates that Google Gemini generally performed consistently across all three metrics, with only slight variability between completeness and relevance. The discrepancy arises because the Friedman test detects overall differences, while the stricter Bonferroni correction may miss smaller pairwise differences, as seen with Google Gemini's metrics.

Effect size calculations using Kendall's W further highlighted these differences. For ChatGPT-4o, Kendall’s W was 0.357, indicating moderate variability across the metrics, suggesting that its performance varied meaningfully across RCL. In contrast, Google Gemini had a Kendall’s W of 0.165, reflecting small variability and more consistent performance across the same metrics. Overall, ChatGPT-4o exhibited greater differences among the evaluated metrics, while Google Gemini maintained steadier performance.

## Discussion

The objective of this study was to address the knowledge gap regarding BCMs by evaluating the performance of two AI platforms. Both ChatGPT-4o and Google Gemini provided high-quality responses, with their overall performance rated as "very good" to "excellent." ChatGPT excelled in the lack of false information, based on raw descriptive statistics, while Google Gemini demonstrated superior relevance with a statistically significant difference. Completeness scores were comparable between the two platforms, and no significant differences were observed in overall performance. However, Google Gemini showed a potential trend of stronger performance in addressing less conventional topics. Variability within metrics was more pronounced for ChatGPT-4o, suggesting differences in how it handles RCL, while Google Gemini exhibited more consistent performance across these metrics. These findings highlight the complementary strengths of the platforms, emphasizing their potential to address gaps in public knowledge about BCMs effectively.

In line with these findings, similar studies across different medical fields have reported consistent patterns of performance between AI platforms. For instance, a study comparing ChatGPT-3.5 and Google Gemini in answering 12 frequently asked pregnancy-related questions found Google Bard (the previous version of Google Gemini) outperforming ChatGPT-3.5 in providing correct and complete responses, with 83% of Bard’s answers rated as acceptable compared to 58% for ChatGPT-3.5. Bard also demonstrated fewer issues with unreliable references compared to ChatGPT-3.5 [11]. Similarly, a study comparing the performance of ChatGPT and Google Gemini on hypertension-related questions supported their use as supplementary tools, highlighting their relevance and minimal errors in addressing patient queries. These findings align with the growing body of evidence suggesting that AI platforms can complement traditional educational resources, such as patient information leaflets, by improving readability and accessibility - even if those leaflets remain superior in completeness and relevance [12].

Further supporting this trend, a study comparing ChatGPT-3.5, ChatGPT-4o, and Google Gemini on glucocorticoid-induced osteoporosis questions found that Google Gemini excelled in providing concise and intuitive responses, while ChatGPT-4 stood out in relevance and guideline adherence [13]. In ophthalmology, ChatGPT-4 and Google Gemini effectively addressed questions about retinal detachment, with ChatGPT-4o demonstrating greater relevance and coherence for complex queries. This highlights the role of these tools in patient education, despite their higher readability demands [14]. Similarly, ChatGPT-4.0 outperformed Google Gemini and Microsoft Copilot in a study evaluating responses to keratoconus-related questions, providing detailed and accurate answers, though its responses were more complex for patients to understand [15]. The application of AI in other domains also demonstrates promising results. For example, ChatGPT-4o outperformed ChatGPT-3.5 in providing primary prevention information for musculoskeletal disorders, scoring higher in completeness, relevance, and appropriateness [16].

Both hypotheses of this study were supported by the results. The overall performance of both platforms was categorized as "Excellent" for most metrics, confirming the first hypothesis. However, Google Gemini significantly outperformed ChatGPT-4o in relevance, as shown by a large effect size and a statistically significant difference, validating the second hypothesis. ChatGPT-4o demonstrated slightly higher performance in the lack of false information compared to Google Gemini, though this difference was not statistically significant. This finding highlights ChatGPT-4o’s potential strength in ensuring factual accuracy but requires further validation in larger datasets to confirm its robustness.

The findings of this study align with prior research comparing AI platforms across various medical fields. For instance, studies evaluating AI platforms in pregnancy-related queries, hypertension management, and glucocorticoid-induced osteoporosis have consistently shown unique strengths for each platform, emphasizing their complementary roles. Similar trends were observed in this study, and Google Gemini’s relevance may make it particularly useful for educational applications, while ChatGPT-4o’s accuracy in avoiding false information ensures reliability in clinical decision-making.

The study also revealed interesting within-model variability. For ChatGPT-4o, completeness and lack of false information differed significantly, highlighting its nuanced performance across metrics. Similarly, Google Gemini demonstrated slight, albeit non-significant, variability between relevance and completeness. These findings underscore the need for continued refinement and further research to optimize the utility of these platforms.

While these tools have shown potential in creating materials when guided by medical experts, they also reveal certain limitations. For instance, a study indicated that ChatGPT responses may be less accurate and transparent compared to traditional methods, like Google Search [23]. One major concern with AI in healthcare is ensuring safety, fairness, and reliability. AI models often reflect biases in their training data and may struggle to generalize to new populations or unfamiliar scenarios, which could lead to inaccurate predictions and potentially harmful outcomes [24]. Additionally, limitations such as outdated information, potential inaccuracies, and ethical concerns around data privacy and accountability highlight the need for cautious use. Overreliance on AI could also undermine critical human judgment and patient interaction [25]. Furthermore, while the AI platforms in this study demonstrated very good to excellent performance, their responses were sometimes complex for ordinary individuals, frequently incorporating medical terminology. This underscores the importance of ensuring that AI systems generate responses in clear, accessible language that adheres to public conversational styles, minimizing technical jargon.

Despite these challenges, AI platforms like ChatGPT continue to demonstrate transformative potential by enhancing patient care, streamlining administrative processes, supporting medical education, facilitating research, and enabling remote consultations. These advancements underscore the importance of using AI as an adjunct to traditional methods, ensuring reliability and safety in clinical applications [25,26]. This study contributes to this growing body of research by highlighting the complementary roles these platforms can play in addressing critical gaps in contraception knowledge.

This study has several limitations that should be acknowledged. First, the scope was limited to evaluating responses from only two AI platforms, ChatGPT-4o and Google Gemini, which may not represent the capabilities of other conversational AI systems. Second, while the questions were systematically selected from a reputable source, the study's design did not incorporate real-world user interaction, which may yield different results due to variability in question phrasing and user behavior. Third, all prompts were presented in Turkish, potentially introducing biases related to language-specific nuances or differences in model training datasets for non-English queries. This study also did not analyze AI performance variations by questioner gender. Additionally, the rubric-based scoring system, while comprehensive, relies on subjective evaluation, which may vary depending on the expertise of the evaluators. Finally, the findings may not be generalizable to broader health topics beyond birth control, as this study focused exclusively on contraception-related questions.

## Conclusions

This study demonstrated the potential of AI platforms to address critical gaps in contraception knowledge. Both ChatGPT-4o and Google Gemini achieved high performance, with complementary strengths: ChatGPT-4o excelling in factual accuracy and Google Gemini demonstrating superior relevance. These findings validate the study’s hypotheses, showing that AI systems can provide reliable, high-quality health information while exhibiting distinct advantages based on their underlying architectures.

The results underscore the value of using these platforms as adjuncts to traditional educational resources, such as web page information from reputable sources and patient information leaflets, rather than standalone tools. By combining the strengths of AI platforms with expert oversight, healthcare providers can improve public health education and accessibility. However, it is essential to address limitations such as biases, lack of generalizability, the use of complex medical terminology in some responses, and ethical concerns around data use. Future research should focus on optimizing AI algorithms, expanding training datasets, and exploring their effectiveness across diverse populations and medical topics. This study contributes to the growing evidence supporting the integration of AI into public health education, emphasizing its potential to complement existing methods and bridge critical knowledge gaps when used responsibly.
